# Aluminum Alloy

**Published:** 1883-11

**Authors:** 


We have received the following from D. G. Harkins, D. D. S., of Springfield, Massachusetts.

ALUMINUM ALLOY.—An eminent chemist of this place has recently produced a new alloy which he thinks will ultimately be used for dental purposes. The fact that a process has been discovered for the rapid and cheap production of aluminum, brings it to the front as a metal destined to play an important part in the industrial world.

This valuable alloy of aluminum is composed of aluminum 10 per cent. pure coppor 90 per cent. It possesses a pale gold color, a hardness surpassing that of bronze, and is susceptible of taking a fine polish. Its hardness and tendency render it peculiarly adopted for various purposess, it can be drawn into wire and its elasticity is an advantage in its favor.

